# Optimized expression of Hfq protein increases *Escherichia coli* growth

**DOI:** 10.1186/s13036-021-00260-x

**Published:** 2021-02-18

**Authors:** Phuong N. L. VO, Hyang-Mi LEE, Jun REN, Dokyun NA

**Affiliations:** Department of Biomedical Engineering, Chung-Ang University, Seoul, 06974 Republic of Korea

**Keywords:** *Escherichia coli*, Growth rate, Cell density, Hfq protein, Expression optimization

## Abstract

*Escherichia coli* is a widely used platform for metabolic engineering due to its fast growth and well-established engineering techniques. However, there has been a demand for faster-growing *E. coli* for higher production of desired substances. Here, to increase the growth of *E. coli* cells, we optimized the expression level of Hfq protein, which plays an essential role in stress responses. Six variants of the *hfq* gene with a different ribosome binding site sequence and thereby a different expression level were constructed. When the Hfq expression level was optimized in DH5α, its growth rate was increased by 12.1% and its cell density was also increased by 4.5%. RNA-seq and network analyses revealed the upregulation of stress response genes and metabolic genes, which increases the tolerance against pH changes. When the same strategy was applied to five other *E. coli* strains (BL21 (DE3), JM109, TOP10, W3110, and MG1655), all their growth rates were increased by 18–94% but not all their densities were increased (− 12 − + 32%). In conclusion, the Hfq expression optimization can increase cell growth rate and probably their cell densities as well. Since the *hfq* gene is highly conserved across bacterial species, the same strategy could be applied to other bacterial species to construct faster-growing strains.

**Supplementary Information:**

The online version contains supplementary material available at 10.1186/s13036-021-00260-x.

## Introduction

The production of chemical substances and proteins from genetically engineered bacteria has been studied for decades for practical use [1], and metabolic engineering has proven the essential role of bacteria engineering and its practical usefulness in the industry [2–4]. For instance, recent advances in metabolic engineering make it possible to engineer bacteria or yeast cells for high and cost-efficient production of recombinant therapeutic proteins such as insulin and growth hormones [5–7] and biofuels such as alcohols, fatty acid methyl, and ethyl esters [8–10]. Regarding recombinant protein production, there have been several strategies to increase protein titers. High copy-number plasmids have been used to produce larger amounts of desired proteins [11, 12], but high copy-number plasmids often place a metabolic burden on host cells, which eventually decreases cell growth, reduces plasmid stability [13], and consequently decreases protein productivity [14, 15]. Optimization of promoter strength has been also used to obtain a high protein titer. Depending on the properties or activities of desired proteins, toxicity, e.g., transcriptional optimization often increases overall protein titers [16–19]. Recently, the increase in bacterial growth rate or cell density has gained great interest to achieve higher production of proteins [20]. Several strategies for improving the cell density of *Escherichia coli* are summarized in Table 1.

**Table 1:** Several studies on improving *E. coli* cell growth

Strategy	Scale	Reference
Alteration in medium components/conditions	Bioreactor/Fermenter	[21, 22]
Regulated membrane dialysis/electro-dialysis reactor/fed-batch fermenter	Bioreactor/Fermenter	[23–27]
Phosphotransacetylase (*pta*)-deleted strain expressing interleukin 2	Fermenter	[28]
Carbon flux redirection to reduce acetate production (introduced *fadR::Tn10* allele into wild-type *E. coli* K-12 VJS632 strain)	Shake flask	[29]
Down-regulated phosphotransacetylase (*pta*) and acetate kinase (*ack*)	Shake flask	[30]
Chimeric GroEL (C-terminal segment from psychrophilic *Pseudoalteromonas* sp. combined with the remainder of GroEL from *E*. *coli*)	N/A	[31]
Co-expression of chaperonins GroEL/GroES	Shake flask	[32]
Random mutagenesis of GroEL/S	96 well plates	[33]

In this study, we developed an *E. coli* strain that had a higher growth rate by optimizing the expression level of Hfq protein. Hfq protein was originally discovered as a host factor for phage Q_β_ (Hfq) replication in *E. coli* [34]. This chaperone, Hfq protein, is an abundant 11 kDa protein that forms a hexameric ring, can be found in more than 50% of bacterial species [35], and present widely in proteobacteria and firmicutes [36, 37]. There have been several studies indicating that Hfq promotes the ability of host cells to resist stresses such as oxidative stress and pH stress [38]. In addition, the role of Hfq in the control of growth-related genes has also been reported [39, 40]. Therefore, we hypothesized that the optimization of Hfq expression could increase bacterial growth rate.

Here, we designed diverse ribosome-binding site (RBS) sequences of the *hfq* gene to fine-tune its expression level by using a computational RBS design tool, RBSDesigner [41], or random screening. A conventional approach to control gene expression in *E. coli* is to use an inducible promoter or a synthetic promoter. The native promoter of *hfq* gene was replaced with an inducible promoter such as *lac* promoter and then its expression level can be controlled by the concentration of its inducer, isopropyl β-d-1-thiogalactopyranoside (IPTG) [42–44]. However, *hfq* transcription is driven from several promoters including three σ^32^-dependent heat shock promoters within the *miaA* open reading frame, P_*mutL*_HS, P_*miaA*_HS, and P1_*hfq*_HS, and four σ^70^-dependent promoters, P_*mutL*_, P_*miaA*_, P2_*hfq*_, and P3_*hfq*_ [45]. Thus, replacement of the *hfq* promoters may result in disruption of cellular regulation systems, and which may lead to unexpected outcomes. Therefore, we designed various RBS sequences to control the level of Hfq protein expression without modifying its promoter. Thereafter, we investigated whether Hfq expression levels affect the growth of various *E. coli* strains.

## Material and methods

### Bacterial strains, plasmids, and antibiotics

Bacterial strains and plasmids used in this study are listed in Table S1. The media used for *E. coli* culture were Luria-Bertani broth (10 g Bacto Tryptone, 5 g yeast extract, and 10 g NaCl per L) [46] and cells were cultured in 250-mL Erlenmeyer flasks at 37 °C shaking incubator. Antibiotic was added to reach the following final concentration: chloramphenicol, 25 μg/mL.

### Various hfq RBS sequences and plasmid construction

Different *E. coli hfq* variants with a variety of translation efficiencies were designed by RBSDesigner. The variants 1–4 were designed by RBSDesigner. To achieve a higher expression level, randomly generated RBS sequences were screened, and variants 5 and 6 were selected. Sequences of primers and genes are shown in Table S2.

Briefly, the amplified *hfq* gene from *E. coli* DH5α strain using HfqF_AatII and HfqR_XhoI primers was cloned into the corresponding restriction sites in pSC101 plasmid. A strong transcriptional terminator T1/TE was cloned downstream of the inserted *hfq* sequence. The *hfq* variant plasmids were constructed by inverse PCR on the pSC-WT *hfq* template. pSC-*hfq-x-*gfp had the *gfp* gene cloned downstream of the Hfq coding sequence using SpeI/XhoI and thereby their coding sequences were fused. To increase the structural flexibility between Hfq and GFP proteins, a stretch of Gly and Ser residues (“GS” linker) was used to connect *hfq* and *gfp* genes. All constructed RBS sequences of the *hfq* variants are listed in Table S3.

### Growth measurement

The *hfq*-harboring cells of *E. coli* DH5α and *hfq*-deleted DH5α were cultured in a 250-mL flask, and their optical densities (OD_600_) were measured every 2 h. The growth of other *E. coli* strains was measured by using Biotek Synergy H1 plate reader (Winooski, VT, USA) at OD_600_ every 1 h for 24 h. All experiments were carried out in triplicate. *E. coli* cells harboring an empty vector were used as a control.

Growth rate (μ) was determined by the equation below [47]:

$$ \mu =\frac{\ln {OD}_2-\ln {OD}_1\ }{{\mathrm{t}}_2-{\mathrm{t}}_1} $$

where (*t*_*2*_ – *t*_*1*_) denotes the interval between two sampling points at *t*_*1*_ and *t*_*2*_, and *OD*_*1*_ and *OD*_*2*_ denote the optical densities of cells sampled at times *t*_*1*_ and *t*_*2*_, respectively.

### Fluorescence measurement

To confirm the expression level of *hfq* variants, the *gfp* gene was cloned downstream of the *hfq* coding sequence as a fusion protein. The fluorescence intensity of GFP was measured by Guava Easycyte HT BG Flow Cytometer (Millipore, Billerica, MA, USA) to determine the expression level of *hfq* variants. For fluorescence measurement, harvest cells were washed and resuspended in 1× phosphate-buffered saline (PBS) buffer at pH 7.4 (Sigma-Aldrich, St. Louis, USA), and subsequently diluted 1000 times with 1× PBS buffer. All experiments were performed in triplicate.

### mRNA-seq analysis

To investigate the effect of *hfq* variants on cellular gene expressions, RNA-seq analysis was conducted. When the *hfq* variant 4 was introduced into *hfq*-deleted DH5α, it achieved the highest cell density and comparably high growth rate. The mRNAs in the cells were prepared for the analysis. mRNAs extracted from *hfq*-deleted DH5α and wild-type DH5α were used as a control. Three samples from individual cultures at stationary phase were used for RNA-seq analysis.

The number of reads for each gene was determined using HTSeq [48]. To reduce gene length bias, Reads Per Kilobase Million (RPKM) of each gene were calculated by dividing the total number of read count aligned to a gene by 1,000,000 and by the length of the gene in kilobase [49].

To identify differentially expressed genes (DEGs), genes were filtered as the following criteria: |log_2_(*fold change*)| > 2; *p*-value < 0.05; and normalized read count ≥ 10. Gene Ontology (GO) and Kyoto Encyclopedia of Genes and Genomes (KEGG) enrichment of DEGs were analyzed by the Database for Annotation, Visualization and Integrated Discovery (DAVID) [50]. Enriched GO terms and KEGG pathways were selected by a *p*-value < 0.05.

The protein-protein interaction (PPI) network of DEGs was constructed using the STRING database [51] and Cytoscape [52]. Highly interconnected clusters were identified using Molecular Complex Detection (MCODE) [53].

## Results and discussion

### Role of Hfq protein in cell growth

There have been reports that the host factor for phage Q_β_ (Hfq) is closely related to cell growth [39]. For example, Hfq protein is associated with stress resistance in *E. coli* [38] and it showed to be crucial for cell survival under nutrient limitation [54]. Hfq protein is also known as a global regulator and is involved in post-transcriptional regulation by facilitating the interaction between small regulatory RNAs (sRNAs) and mRNAs [55, 56], and regulations of RNA stability [57, 58]. Hfq protein also controls the activity of several proteins involved in mRNA turnover by directly or indirectly interacting with RNase E [59], polynucleotide phosphorylase, and poly(A) polymerase [60–63]. Therefore, we hypothesized that increased Hfq protein expression may promote cell growth by enhancing resistance to environmental stresses. However, since over-expression of Hfq protein may disrupt cellular physiology by extensive protein expression alteration, which may retard cell growth, we also hypothesized that the expression of Hfq protein should be finely optimized.

### Construction of various RBS sequences to diversify the expression level of Hfq protein in *E. coli*

**Fig. 1:**
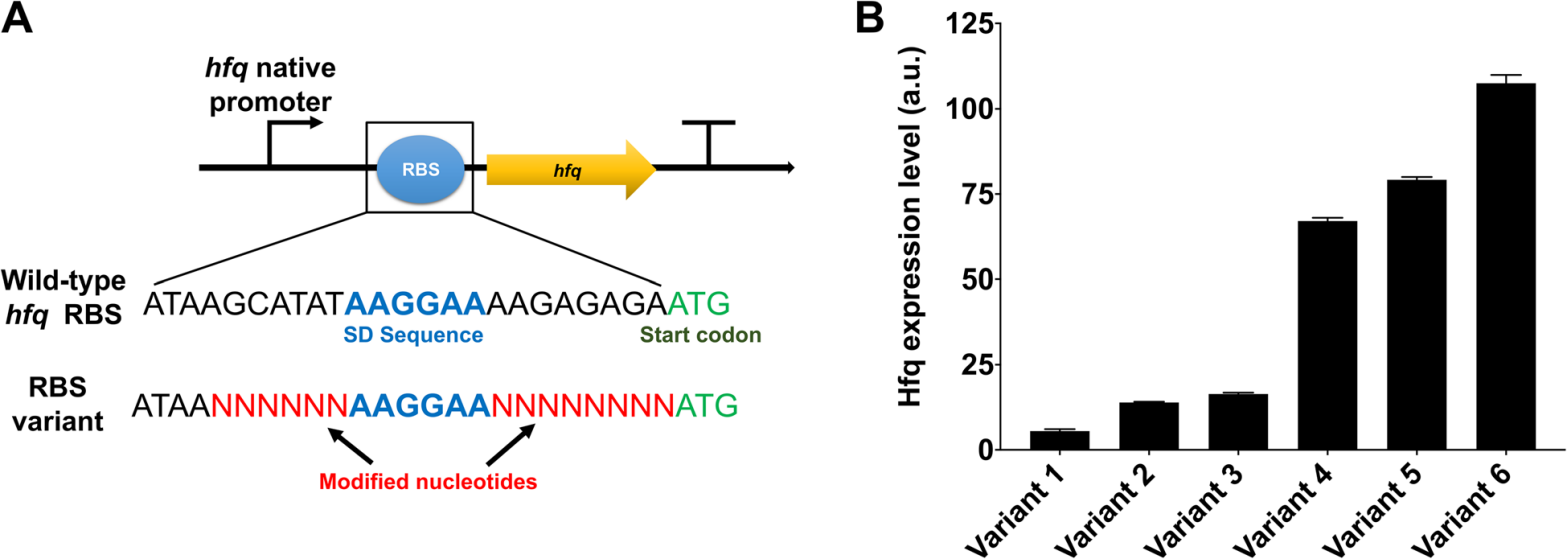
Constructed *hfq* variants and their expression levels. **a** Constructed *hfq* gene structure and RBS variants. Four to six nucleotides upstream of Shine Dalgarno (SD) sequence and eight nucleotides downstream of SD designed or randomly generated. To avoid prolonged transcription downstream of the *hfq* gene, a transcription terminator was added to the gene construct. **b** GFP-fused Hfq expression levels. The green fluorescence intensity emitted from *E*. *coli* DH5α with a *hfq* variant was measured. Three replicates and mean ± SD. When tested with each other by t-test, there were no statistically significant differences only between the expression levels of variants 2 and 3 (*p*-value < 0.05)

We constructed six RBS variants of the *hfq* gene by a computational model and random screening (Table S3) to achieve a desired expression level of Hfq protein (Fig. 1a) and thereby increase cell growth. To avoid the disruption of inherent regulation of *hfq* transcription, its native promoters were used without modification (Fig. 1a). To maintain a low copy number of the *hfq* gene, the constructed genes were introduced into the pSC101 plasmid with only < 5 copies in *E. coli*.

To confirm the expression levels of the constructed *hfq* variants, the GFP coding sequence was fused at the C-terminus of the *hfq* coding sequence with a stretch of Gly and Ser residues (GS linker). The constructed variants show diverse expression levels of Hfq protein (Fig. 1b). The cells harboring *hfq* variant 6 displayed the highest expression level, whereas *hfq* variant 1 demonstrated the lowest expression level, which was 19.5-fold lower than that of *hfq* variant 6.

### Fine optimization of Hfq expression increased *E. coli* growth

**Fig. 2:**
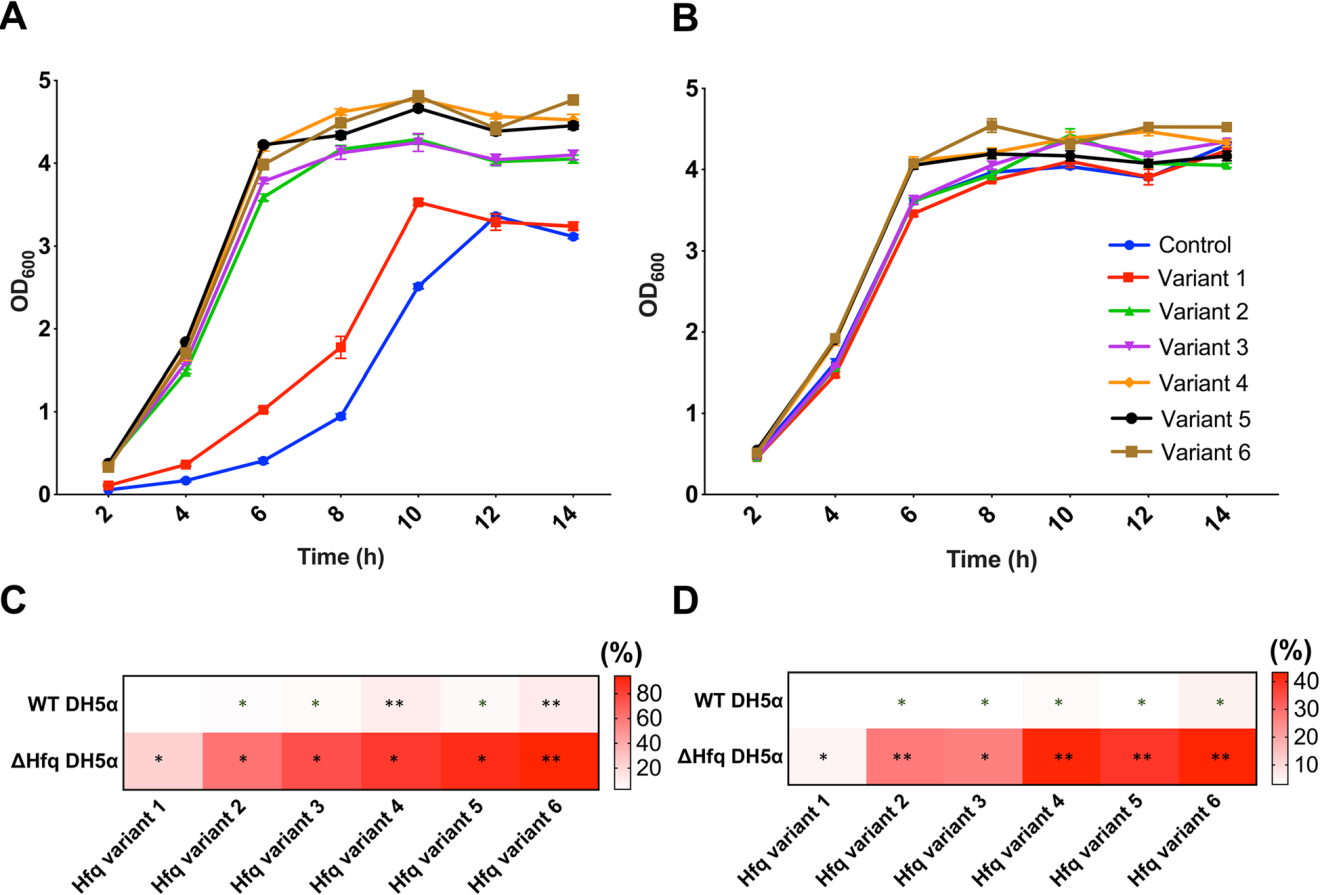
The effect of diverse *hfq* variants on the growth of wild-type DH5α and *∆hfq* DH5α cells. Growth curves of *∆hfq* DH5α cells (**a**) and wild-type DH5α cells (**b**) when an *hfq* variant was introduced. Relative growth rate increase (**c**) and relative cell density increase (**d**) by the *hfq* variants. To calculate the relative values, the measured values were compared with that of control cells that did not harbor any *hfq* variants. * denotes *p*-value < 0.05 and ** denotes *p*-value < 0.0001

To effectively investigate the difference in cell growth, the *hfq* variants were introduced into *hfq*-deleted DH5α cells (Fig. 2a). For comparison, the variants were also introduced into wild-type DH5α cells (Fig. 2b). As shown in Fig. 2a, deletion of *hfq* gene dramatically reduced growth rate and maximum cell density (optical density measured at 600 nm): the measured growth rate was 0.422 ± 0.005 h^− 1^ and the highest OD was 3.36. Introduction of the *hfq* variants recovered the growth rate as well as maximum cell density. Variant 4 achieved the highest cell density (42.2% increase) in *hfq*-deleted DH5α cells. Though the variant 6 achieved the highest growth rate (94.3% increase), the variant 4 also achieved a comparably high growth rate (83.4% increase) (Fig. 2c and d). Regarding wild-type DH5α cells, since the cells already harbor an inherent *hfq* gene, the *hfq* variants could not significantly increase growth (Fig. 2b, c, and d). The growth rate and maximum OD of wild-type DH5α cells were 0.587 ± 0.001 h^− 1^ and 4.47, respectively. When Hfq expression was optimized in wild-type DH5α cells, the growth rate was increased by 12.1% by the variant 6 and the cell density was increased only by 4.5% by the same variant. These results represent that Hfq expression optimization is able to increase cell growth.

### Differentially expressed genes

To investigate the biological effect of Hfq expression on cellular physiology leading to the increase in bacterial growth, we performed RNA-seq analysis to discover up- and down-regulated genes of the three strains (*hfq* variant 4 in *hfq*-deleted DH5α, *hfq*-deleted DH5α, and wild-type DH5α). Hfq protein is an RNA-binding protein, specifically interacts with sRNAs to regulate mRNAs at post-transcription stage. Thus, RNA-seq analysis does not reveal the direct relationship between Hfq protein and transcripts. However, since transcripts encoding for proteins are actual players in cellular physiology, RNA-seq approach is useful to capture which cellular functions are implicated in increased cell growth.

Firstly, we compared the up- and down-regulated genes between *hfq* variant 4 in *hfq*-deleted DH5α and *hfq*-deleted DH5α. It was expected that *hfq*-deleted cells were an effective control to clearly reveal the underlying mechanisms related to improved cell growth. However, as explained in Text S1, the absence of the global regulator, Hfq protein, globally affected the cellular expression of genes even that are not related to cell growth, and as a result the RNA-seq analysis results misled to an inappropriate conclusion (Fig. S1).

**Fig. 3:**
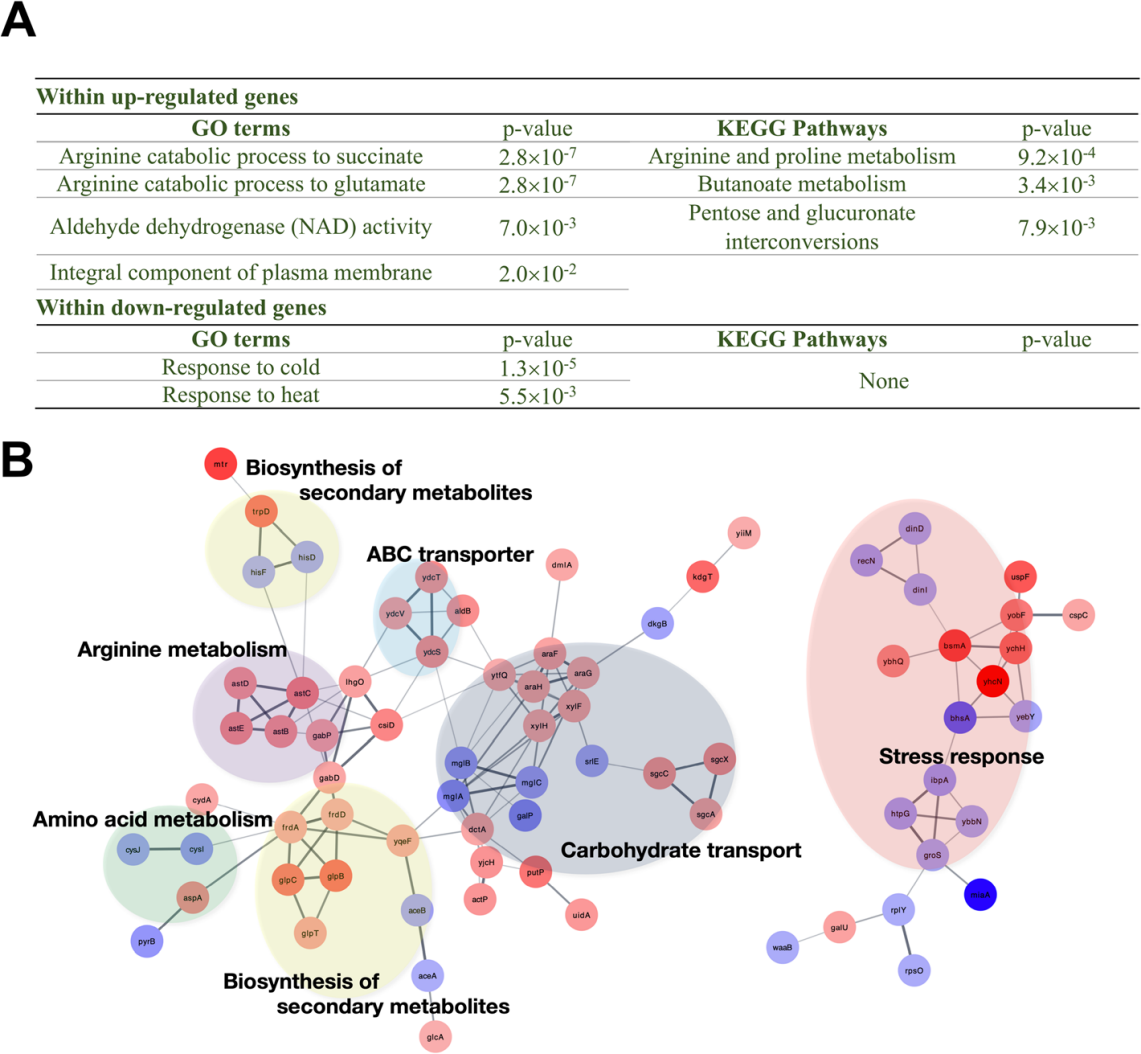
RNA-seq analysis results including enriched annotations and dense clusters. (**a**) Enriched GO terms and KEGG pathways within the DEGs. (**b**) Dense clusters constructed from the DEGs. Red and blue colors denote up- and down-regulated genes, respectively

Thus, we compared the expression patterns of *hfq* variant 4 in *hfq*-deleted DH5α and wild-type DH5α. The *hfq*-deleted DH5α cells harboring variant 4 showed a higher growth rate as well as a higher cell density than wild-type DH5α, and therefore wild-type DH5α cells already expressing the inherent *hfq* gene would be a better control to eliminate the global effect of Hfq protein. From the RNA-seq analysis, we found 110 differentially expressed genes (DEGs) with |log_2_(*fold change*)| > 2 and *p*-value < 0.05. Of the 110 genes, 64 genes were up-regulated and the remaining 46 genes were down-regulated. We performed enrichment analysis of GO terms and KEGG pathways within the DEGs to investigate physiological differences between variant 4 and wild-type DH5α *E. coli*. The DAVID bioinformatics tool [50] was utilized to identify the functions enriched within DEGs. The enriched terms are listed in Fig. 3a. In addition to the enrichment analyses, we generated a protein-protein interaction network of the DEGs using STRING database [64] and identified densely interconnected clusters. As shown in Fig. 3b, six highly interconnected clusters were identified by using Cytoscape [52] and MCODE [53]. The clusters were functionally similar to the enriched GO terms and KEGG pathways.

As bacterial cells grow in LB broth, alkalinization occurs and cells are exposed to alkaline stress (Fig. S1C) [46]. Compared with wild-type *E. coli*, the cells harboring the variant 4 showed up-regulated biological processes such as “*Arginine catabolic process to succinate*” (GO:0019545, *p*-value = 2.79 × 10^− 7^), “*Arginine catabolic process to glutamate*” (GO:0019544, *p*-value = 2.79 × 10^− 7^), and “*Arginine and proline metabolism*” (eco00330, *p*-value = 9.23 × 10^− 4^). These processes convert molecules into acids instead of producing alkaline amines, and which could reduce the alkaline stress. Additionally, of the three up-regulated genes (*aldB*, *gabD*, and *astD*) involved in “*Aldehyde dehydrogenase (NAD) activity*” (GO:0004029, *p*-value = 7.0 × 10^− 3^), *gabD* encodes for a succinic semialdehyde dehydrogenase (SSDH) that plays an important role in γ-aminobutyric acid (GABA) shunt pathway to produce succinate. The *astD* encodes for succinylglutamic semialdehyde dehydrogenase involved in the arginine catabolism to glutamate. Aldehyde dehydrogenase B (*aldB*) was previously reported as an NADP^+^-dependent general detoxifying enzyme [65]. Besides, arginine is an important carbon and nitrogen source during starvation (stationary phase) [66], and the production of glutamate and succinate also fuels the tricarboxylic acid cycle (TCA) for energy generation.

The processes “*Butanoate metabolism*” (eco00650, *p*-value = 3.4 × 10^− 3^) and “*Pentose and glucuronate interconversions*” (eco00040, *p*-value = 7.9 × 10^− 3^) are carbohydrate metabolism pathways in *E. coli*. All the genes involved in “*Butanoate metabolism*” encode for the enzymes that catalyze the synthesis of metabolites such as fumarate (catalyzed by *frdA, frdD, gabD*), pyruvate (catalyzed by *dmlA*), and acetyl-CoA (catalyzed by *yqeF*), which eventually enter the TCA cycle. “*Pentose and glucuronate interconversions*” are responsible for the production of L-xylonate and *L*-*lyxonate* (non-*oxidative* branch)*,* degradation of L-arabinose to form D-xylulose-5-phosphate, eventually entering the pentose phosphate pathway (PPP). The PPP is one of the primary sources for the generation of NADPH (reduced nicotinamide adenine dinucleotide phosphate), which is an essential electron donor, and serves as the reducing power for the biosynthesis of all major cell components [67, 68]. In addition, several processes related to cold and heat shock responses were down-regulated in the cells harboring the variant 4: “*Response to cold*” (GO:0009409, *p*-value = 1.3 × 10^− 5^) and “*Response to heat*” (GO:0009408, *p*-value = 5.5 × 10^− 3^). Since the cells were grown at an isothermal condition, 37 °C, the genes involved in temperature-dependent processes seemed to be down-regulated for better performance of cells.

Overall, GO and KEGG enrichment analyses and network analysis indicate that the variant 4 increased the growth of *E. coli* by producing more acidic molecules to reduce alkaline stress and by directing nutrient sources to the TCA cycle to generate energy and provide metabolites to essential pathways.

### Application of *hfq* variants to promote the growth of other *E. coli* strains

In this study, optimization of Hfq protein expression was able to increase cell growth and it opens a new way to enhance bacterial cell growth. To prove the practical applicability of the strategy, we applied the constructed variants to optimize Hfq expression in various *E. coli* strains: one B strain (BL21 (DE3)) and four K12-derivative strains (JM109, TOP10, W3110, and MG1655). The B strain is widely used in industry to produce proteins, and the K12-derivative strains are commonly used for molecular biology studies.

**Fig. 4:**
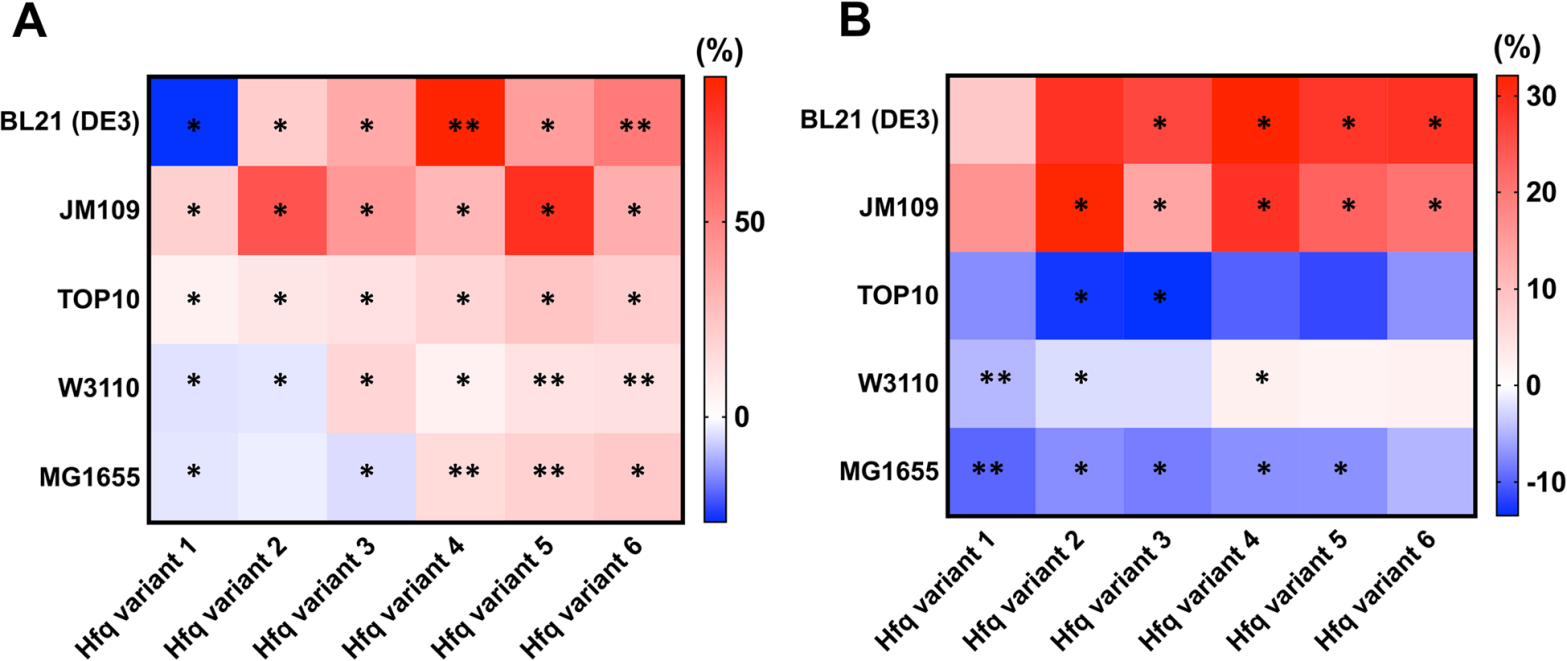
Cell density and growth rate of various *E. coli* strains harboring an *hfq* variant. The six *hfq* variants were introduced into the five wild-type *E. coli* strains and their relative increases of growth rate (**a**) and cell density (**b**) are shown. The relative values denote the percent increase or decrease compared with respective control cells harboring no *hfq* variants. * denotes *p*-value < 0.05 and ** denotes *p*-value < 0.0001

Hfq expression optimization was able to improve growth rate in all strains (Fig. 4a), but not all strains showed improved cell density by the variants (Fig. 4b). For example, the growth rate of the industrial strain BL21 (DE3) was improved by 87.2% by variant 4 and this variant also increased cell density by 32.1%. For JM109, the variants 2 and 4 increased a growth rate by 67.2 and 80.6%, and also increased a cell density by 31.6 and 22.9%, respectively. For W3110, only variants of a higher expression level could enhance both cell growth rate and density. For MG1655, the variant 6 increased a growth rate by 23.1%, but its cell density was decreased by 4.8%. For TOP10 and MG1655, their cell densities were decreased by the variants though their growth rates were improved.

## Conclusion

This study proves a strong correlation between Hfq expression and *E. coli* growth and that the optimization of Hfq expression improves *E. coli* growth via regulation of several pathways participating in cellular stress tolerance. Since the *hfq* protein is widely conserved in bacterial species, the strategy could be applied to other bacterial strains to enhance cell growth. This approach would be beneficial for constructing cell factories with slow-growing bacteria.

## Supplementary Information

**Additional file 1: Table S1.** Bacterial strains and plasmids used in this study. **Table S2.** Oligonucleotide primers and PCR templates used in this study. **Table S3.** Constructed *hfq* variants. **Text S1.** RNA-seq analysis results when *hfq*-deleted cell was used as a control. **Figure S1.** RNA-seq analysis results and investigation of the association of acid resistance with cell growth.
