# Supplementary material for: Optimized expression of Hfq protein increases Escherichia coli growth
Source: J Biol Eng. 2021 Feb 18;15:7. doi: 10.1186/s13036-021-00260-x (PMC7890833; doi:10.1186/s13036-021-00260-x)
Supplement: Supplementary file 1 — Additional file 1: Table S1. Bacterial strains and plasmids used in this study. Table S2. Oligonucleotide primers and PCR templates used in this study. Table S3. Constructed hfq variants. Text S1. RNA-seq analysis results when hfq-deleted cell was used as a control. Figure S1. RNA-seq analysis results and investigation of the association of acid resistance with cell growth. [file 13036_2021_260_MOESM1_ESM.docx]

**Supplementary Information**

**Optimized expression of Hfq protein increases *Escherichia coli* growth**

Phuong N. L. VO, Hyang-Mi LEE, Jun REN, and Dokyun NA

1. Table S1. Bacterial strains and plasmids used in this study.
2. Table S2. Oligonucleotide primers and PCR templates used in this study.
3. Table S3. Constructed *hfq* variants.
4. Text S1. RNA-seq analysis results when *hfq*-deleted cell was used as a control.
5. Figure S1. RNA-seq analysis results and investigation of the association of acid resistance with cell growth.

**Table S1.** Bacterial strains and plasmids used in this study.

| **Strains/plasmids** | **Characteristics** | **Reference** |
| --- | --- | --- |
| **Strains** |  |  |
| *E*. *coli* DH5α | F^–^ *endA1* *glnV44* *thi-1 recA1* *relA1* *gyrA96*  *deoR* *nupG* *purB20* φ80d*lacZ*ΔM15 Δ(*lacZYA-argF*)U169, hsdR17(*r_K_*^–^*m_K_*^+^), λ^–^ | Invitrogen |
| *E*. *coli* BL21 (DE3) | *E. coli str. B F^–^ ompT gal dcm lon hsdS_B_(r_B_^–^m_B_^–^) λ(DE3 [lacI lacUV5-T7p07 ind1 sam7 nin5]) [malB^+^]_K-12_(λ^S^)* | NEB |
| *E*. *coli* JM109 | *endA1 glnV44 thi-1 relA1 gyrA96 recA1 mcrB^+^ Δ(lac-proAB) e14- [*F' *traD36 proAB^+^ lacI^q^ lac*ZΔM15] hsdR17(*r_K_^-^m_K_*^+^) | NEB |
| *E*. *coli* TOP10 | F^–^ *mcrA Δ(mrr-hsdRMS-mcrBC) φ80lac*ZΔM15 *ΔlacX74 nupG recA1 araD139 Δ(ara-leu)7697 galE15 galK16 rpsL(Str^R^) endA1 λ^-^* | Invitrogen |
| *E*. *coli* W3110 | F^–^*λ^-^ rph-1 INV(rrnD, rrnE)* | NEB |
| *E*. *coli* MG1655 | K-12 F^–^ λ^–^ *ilvG*^–^ *rfb-50* *rph-1* | NEB |
| *E*. *coli* ∆*hfq*-DH5α | F^–^ φ80*lacZ*ΔM15 Δ(*lacZ*YA-*arg*F)U169 *rec*A1 *end*A1 *hsd*R17(r_K_^–^, m_K_^+^) *pho*A *sup*E44 *λ*^–^ *thi*-1 *gyr*A96 *rel*A1 ∆*hfq* | In this study |
| **Plasmids** |  |  |
| pSC101 | psc101 replicon, Cm^R^ | In this study |
| psc101-Amp | psc101 replicon, Amp^R^ | In this study |
| pSC-WT *hfq* | Containing WT *hfq* PCR fragment from *E*. *coli* DH5α, T1/TE, psc101 replicon, Cm^R^ | In this study |
| pA-*gfp* | p15A replicon, lac promoter, *gfp*, T1/TE, Km^R^ | In this study |
| ***hfq* variants** |  |  |
| pSC-*hfq* variant 1 | Containing *hfq* variant 1, T1/TE, psc101 replicon, Cm^R^ | In this study |
| pSC-*hfq* variant 2 | Containing *hfq* variant 2, T1/TE, psc101 replicon, Cm^R^ | In this study |
| pSC-*hfq* variant 3 | Containing *hfq* variant 3, T1/TE, psc101 replicon, Cm^R^ | In this study |
| pSC-*hfq* variant 4 | Containing *hfq* variant 4, T1/TE, psc101 replicon, Cm^R^ | In this study |
| pSC-*hfq* variant 5 | Containing *hfq* variant 5, T1/TE, psc101 replicon, Cm^R^ | In this study |
| pSC-*hfq* variant 6 | Containing *hfq* variant 6, T1/TE, psc101 replicon, Cm^R^ | In this study |
| ***hfq* variants-*gfp*** |  |  |
| pSC-WT *hfq-gfp* | Containing WT *hfq* fused to *gfp* by (GS)_n_ linker, T1/TE, psc101 replicon, Cm^R^ | In this study |
| pSC-*hfq1-gfp* | Containing *hfq* variant 1 fused to *gfp* by (GS)_n_ linker, T1/TE, psc101 replicon, Cm^R^ | In this study |
| pSC-*hfq2-gfp* | Containing *hfq* variant 2 fused to *gfp* by (GS)_n_ linker, T1/TE, psc101 replicon, Cm^R^ | In this study |
| pSC-*hfq3-gfp* | Containing *hfq* variant 3 fused to *gfp* by (GS)_n_ linker, T1/TE, psc101 replicon, Cm^R^ | In this study |
| pSC-*hfq4-gfp* | Containing *hfq* variant 4 fused to *gfp* by (GS)_n_ linker, T1/TE, psc101 replicon, Cm^R^ | In this study |
| pSC-*hfq5-gfp* | Containing *hfq* variant 5 fused to *gfp* by (GS)_n_ linker, T1/TE, psc101 replicon, Cm^R^ | In this study |
| pSC-*hfq6-gfp* | Containing *hfq* variant 6 fused to *gfp* by (GS)_n_ linker, T1/TE, psc101 replicon, Cm^R^ | In this study |

**Table S2.** Oligonucleotide primers and PCR templates used in this study.

| **Constructs** | **Template** | **Oligo names** | **Oligonucleotides (5’ – 3’)** |
| --- | --- | --- | --- |
| pSC-WT *hfq* | *E*. *coli* DH5α | HfqF_AatII  HfqR_XhoI | ATGCGACGTCGGCGTTAGCCATTGAGCTG  ATGCCTCGAGCCGTGTAAAAAAACAGCCCG |
| pSC-*hfq* variant 1 | pSC-WT *hfq* | HFQ_R  1-000185F | TTGTACTTTGAACCTTTCGATTCTG  ATAAGCCCTGAAGGAAAATCACAAATGGCTAAGGGGCAATCTTTAC |
| pSC-*hfq* variant 2 | pSC-WT *hfq* | HFQ_R  2-0011F | TTGTACTTTGAACCTTTCGATTCTG  ATAAGCCGTGAAGGAAAATCACAAATGGCTAAGGGGCAATCTTTAC |
| pSC-*hfq* variant 3 | pSC-WT *hfq* | HFQ_R  3-0101F | TTGTACTTTGAACCTTTCGATTCTG  ATAAGCATATAAGGAACACGCGGAATGGCTAAGGGGCAATCTTTAC |
| pSC-*hfq* variant 4 | pSC-WT *hfq* | HFQ_R  4-105F | TTGTACTTTGAACCTTTCGATTCTG  ATAAGCTTTAAAGGAACAAATTTTATGGCTAAGGGGCAATCTTTAC |
| pSC-*hfq* variant 5 | pSC-WT *hfq* | HFQ_R  5-003F | TTGTACTTTGAACCTTTCGATTCTG  ATAAATTGATAAGGAAAAGAGAGAATGGCTAAGGGGCAATCTTTAC |
| pSC-*hfq* variant 6 | pSC-WT *hfq* | HFQ_R  6-0045F | TTGTACTTTGAACCTTTCGATTCTG  ATAAATTGAGAAGGAAAAGAGAGAATGGCTAAGGGGCAATCTTTAC |

**Table S3.** Constructed *hfq* variants.

| ***E*. *coli* DH5α**  WT-*hfq* | **Sequence** |
| --- | --- |
|  | ATAAGCATAT**AAGGAA**AAGAGAGAatg |
|  |  |
| **Variant *hfq*** |  |
| *hfq* variant 1 | ATAAGCCCTG**AAGGAA**AATCACAAatg^*^ |
| *hfq* variant 2 | ATAAGCCGTG**AAGGAA**AATCACAAatg |
| *hfq* variant 3 | ATAAGCATAT**AAGGAA**CACGCGGAatg |
| *hfq* variant 4 | ATAAGCTTTA**AAGGAA**CAAATTTTatg |
| *hfq* variant 5 | ATAAATTGAT**AAGGAA**AAGAGAGAatg |
| *hfq* variant 6 | ATAAATTGAG**AAGGAA**AAGAGAGAatg |

* Bold nucleotides denote Shine-Dalgarno (SD) sequences, red nucleotides denote modified nucleotides.

**Text S1. RNA-seq analysis results when *hfq*-deleted cell was used as a control**

To investigate the biological effect of Hfq expression on cellular physiology leading to the increase in bacterial growth, we performed RNA-seq analysis to discover up- and down-regulated genes of the two strains (*hfq* variant 4 in *hfq*-deleted cells vs. *hfq*-deleted cells as a control). It was expected that the use of *hfq*-deleted cells as a control could allow us to clearly capture the altered genes and cellular processes affected by the *hfq* variant. However, since Hfq protein is a global regulator, many genes affected by Hfq protein but not associated with cell growth were identified, which misled us to biased interpretation.

Briefly, we found 446 DEGs with a |log_2_ (*fold-change*)| > 2 and *p*-value < 0.05. Of the 446 genes, 200 genes were up-regulated, and the remaining 246 genes were down-regulated. We performed enrichment analyses of GO terms and KEGG pathways to determine physiological differences between variant 4 and *∆hfq E*. *coli*. DAVID bioinformatics tool (1) was used to identify the functions enriched within the DEGs. The enriched terms are listed in Figure S1A. To identify the core functional modules in the DEGs, we mapped the DEGs onto the PPI network obtained from the STRING database (2). Four highly interconnected sub-networks were identified by using Cytoscape (3) and MCODE (4). Three of the modules were composed of down-regulated genes, and one module was mostly composed of up-regulated ones (Fig. S1B).

The up-regulated modules included proteins mostly involved in the acid resistance system of *E*. *coli* (5): *dctR*, *gadA*, *gadB*, *gadC*, *gadE*, *gadW*, *gadX*, *hdeA*, *hdeB*, *hdeD*, *slp*, and *yhiD*. HdeA, HdeB, and HdeD are known as acid-resistance-related proteins. Furthermore, the sub-network included the glutamate decarboxylase system (*gad* regulon) (6, 7). GadA and GadB encode for isozymes of glutamate decarboxylases catalyzing the conversion of glutamate to γ-aminobutyrate. GadC encodes a putative glutamate:γ-aminobutyrate antiporter (8). GadE, GadX, and GadW were identified as a transcriptional activator of glutamate decarboxylase genes, and plays an essential role in the glutamate-dependent acid resistance (GDAR) system (9-13). Several studies have reported that proved the important role of the glutamate decarboxylase system or the protection of *E*. *coli* against acidic environment, or metabolite stress in high-density cells (12-14).

In order to investigate the association of acid tolerance with cell growth, the pH change of media was monitor over time (Fig. S1C). Interestingly, the media was alkalized because amino acids in LB media were catabolized by bacterial cells (15), which is inconsistent with the RNA-seq analysis result. To confirm the non-association of acid tolerance, the *hfq* variant 4-containing cells were incubated at low pH (pH = 2.5) and the cells showed a remarkable decrease in acid tolerance (Fig. S1D). Since Hfq protein is a global regulator, wide range of genes were affected by Hfq protein and many of the altered genes may not be associated cell growth and which biased the analysis and consequently misled to an inappropriate hypothesis.

**Figure S1. RNA-seq analysis results and investigation of the association of acid resistance with cell growth**

(A) Enriched GO terms and KEGG pathways identified from the DEGs. (B) Dense protein interaction clusters identified from the DEGs. (C) pH change of LB media over time. (D) Survival rates of wild-type cells and *hfq*-deleted cells harboring the variant 4 at low pH (pH = 2.5). At 10 min post-incubation, the survival rates were significantly different (*p*-value < 0.05), but at other time points there were no significant differences.

**References**

1. Huang da W, Sherman BT, Lempicki RA. Systematic and integrative analysis of large gene lists using DAVID bioinformatics resources. Nat Protoc. 2009;4(1):44-57.

2. Szklarczyk D, Franceschini A, Wyder S, Forslund K, Heller D, Huerta-Cepas J, et al. STRING v10: protein-protein interaction networks, integrated over the tree of life. Nucleic Acids Res. 2015;43(Database issue):D447-452.

3. Shannon P, Markiel A, Ozier O, Baliga NS, Wang JT, Ramage D, et al. Cytoscape: a software environment for integrated models of biomolecular interaction networks. Genome Res. 2003;13(11):2498-2504.

4. Bader GD, Hogue CW. An automated method for finding molecular complexes in large protein interaction networks. BMC Bioinformatics. 2003;4:2.

5. Masuda N, Church GM. Regulatory network of acid resistance genes in *Escherichia coli*. Mol Microbiol. 2003;48(3):699-712.

6. Lin J, Smith MP, Chapin KC, Baik HS, Bennett GN, Foster JW. Mechanisms of acid resistance in enterohemorrhagic *Escherichia coli*. Appl Environ Microbiol. 1996;62(9):3094-3100.

7. Castanie-Cornet MP, Penfound TA, Smith D, Elliott JF, Foster JW. Control of acid resistance in *Escherichia coli*. J Bacteriol. 1999;181(11):3525-3235.

8. Hersh BM, Farooq FT, Barstad DN, Blankenhorn DL, Slonczewski JL. A glutamate-dependent acid resistance gene in *Escherichia coli*. J Bacteriol. 1996;178(13):3978-3981.

9. Hommais F, Krin E, Coppee JY, Lacroix C, Yeramian E, Danchin A, et al. GadE (YhiE): a novel activator involved in the response to acid environment in *Escherichia coli*. Microbiology. 2004;150:61-72.

10. Tramonti A, De Canio M, Delany I, Scarlato V, De Biase D. Mechanisms of transcription activation exerted by GadX and GadW at the *gadA* and *gadBC* gene promoters of the glutamate-based acid resistance system in *Escherichia coli*. J Bacteriol. 2006;188(23):8118-8127.

11. Castanie-Cornet MP, Cam K, Bastiat B, Cros A, Bordes P, Gutierrez C. Acid stress response in *Escherichia coli*: mechanism of regulation of gadA transcription by RcsB and GadE. Nucleic Acids Res. 2010;38(11):3546-3554.

12. De Biase D, Pennacchietti E. Glutamate decarboxylase-dependent acid resistance in orally acquired bacteria: function, distribution and biomedical implications of the *gadBC* operon. Mol Microbiol. 2012;86(4):770-786.

13. Seo SW, Kim D, O'Brien EJ, Szubin R, Palsson BO. Decoding genome-wide GadEWX-transcriptional regulatory networks reveals multifaceted cellular responses to acid stress in *Escherichia coli*. Nat Commun. 2015;6:7970.

14. Mates AK, Sayed AK, Foster JW. Products of the *Escherichia coli* acid fitness island attenuate metabolite stress at extremely low pH and mediate a cell density-dependent acid resistance. J Bacteriol. 2007;189(7):2759-2768.

15. Sezonov G, Joseleau-Petit D, D'Ari R. *Escherichia coli* physiology in Luria-Bertani broth. J Bacteriol. 2007;189(23):8746-8749.
